# The impact of population size on the evolution of asexual microbes on smooth versus rugged fitness landscapes

**DOI:** 10.1186/1471-2148-9-236

**Published:** 2009-09-18

**Authors:** Andreas Handel, Daniel E Rozen

**Affiliations:** 1 Department of Epidemiology and Biostatistics, College of Public Health, University of Georgia, Athens, GA 30602, USA; 2 University of Manchester, Manchester, M13 9PT, UK

## Abstract

**Background:**

It is commonly thought that large asexual populations evolve more rapidly than smaller ones, due to their increased rate of beneficial mutations. Less clear is how population size influences the level of fitness an asexual population can attain. Here, we simulate the evolution of bacteria in repeated serial passage experiments to explore how features such as fitness landscape ruggedness, the size of the mutational target under selection, and the mutation supply rate, interact to affect the evolution of microbial populations of different sizes.

**Results:**

We find that if the fitness landscape has many local peaks, there can be a trade-off between the rate of adaptation and the potential to reach high fitness peaks. This result derives from the fact that whereas large populations evolve mostly deterministically and often become trapped on local fitness peaks, smaller populations can follow more stochastic evolutionary paths and thus locate higher fitness peaks. We also find that the target size of adaptation and the mutation rate interact with population size to influence the trade-off between rate of adaptation and final fitness.

**Conclusion:**

Our study suggests that the optimal population size for adaptation depends on the details of the environment and on the importance of either the ability to evolve rapidly or to reach high fitness levels.

## Background

Understanding the factors that influence the evolution of microbial populations not only provides fundamental insights into evolutionary processes [1-4], but is also of considerable applied importance, owing to the fact that many microbes are pathogenic. Development of a predictive framework of microbial evolutionary dynamics is central to understanding processes such as the evolution of drug resistance [5-7] and the emergence of novel infectious diseases [8,9]. Numerous interacting factors determine evolutionary patterns of microbes, but all are likely influenced by the size of the microbial population. In this work we focus our attention on the consequences of population size in asexual microbes, and study how changes in this parameter interact with other factors to modify its role and importance in adaptive dynamics.

**Figure 1:**
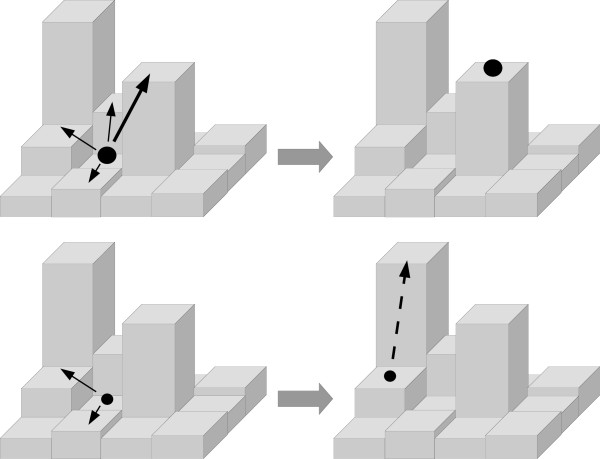
**Schematic evolution of large and small asexual populations**. Top panels: A large population quickly generates all possible beneficial 1-step mutations, the fittest of which (represented by the thick arrow) is most likely to become fixed. In this example, the newly fixed mutant only has access to deleterious mutations that reduce fitness, it is trapped on a local fitness peak. Bottom panels: Small populations have reduced access to beneficial mutations. This can lead to the fixation of a mutant with intermediate fitness, which has access to an even higher fitness peak (dashed arrow). This allows the small population to eventually reach a higher level of fitness than the large population, albeit at a slower speed.

It is widely believed that the higher supply of beneficial mutations allows large asexual populations to adapt more rapidly to new environments compared to small populations [10-13]. However, the speed at which adaptation occurs is only one component of evolutionary dynamics. Another important component is the magnitude of fitness obtained following the adaptive process. The latter component can result in the reduction or elimination of an adaptive advantage enjoyed by large asexual populations if the fitness landscape on which evolution occurs is a rugged one that contains many local peaks [14]. This result is explained as follows: If a population is large, its members can fully sample all possible 1-step beneficial mutations from a given genotype. Such large populations will tend to become fixed for the beneficial mutations carrying the largest benefits in an almost deterministic fashion, a process enabling the large population to reach the nearest fitness peak quite rapidly. If this peak represents a local optimum, large asexual populations may become trapped there, unable to reach a global optimum (Figure 1 top).

On the other hand, a small population will generate only a subset of all possible 1-step beneficial mutations, with few mutations that confer large fitness effects [13,15,16]. Both the reduced supply rate of new mutations and their smaller fitness benefits contribute to the expected slower rate of adaptation of small versus large populations. However, at the same time the small populations will follow more stochastic adaptive trajectories [14], and this increases their ability to explore the more distant fitness landscape. With this broader exploration comes an increased likelihood of reaching more distant and higher fitness peaks (Figure 1 bottom). Thus while both large and small populations can become trapped upon local optima, small populations may be more able to avoid this trap and consequently reach higher fitness peaks. In the present work, we use computer simulations to explore this phenomenon in more detail, focusing on factors that might modify the role of population size during adaptive evolution. In particular, we focus on the interaction of population size with factors that are likely to influence the adaptive trajectories of microbes; namely ruggedness of the fitness landscape, the target size of adaptation and mutation rates.

## The model

We simulate the evolution of bacteria as they undergo repeated cycles of growth and serial dilution [1,14]. At the start of each simulation, the population consists of *N*_0 _identical clones. The bacteria go through *D *rounds of division, and each bacterium produces offspring depending on fitness, *f*, as 2^*f*^. After *D *divisions, serial transfer, modeled as multinomial sampling, reduces the population size back to *N*_0 _which initiates another round of exponential growth. This procedure is iterated until the desired number of generations is reached. Because bacterial death is ignored, the only way a given clone can be eliminated is if it is not sampled during serial transfer [17,18].

**Figure 2:**
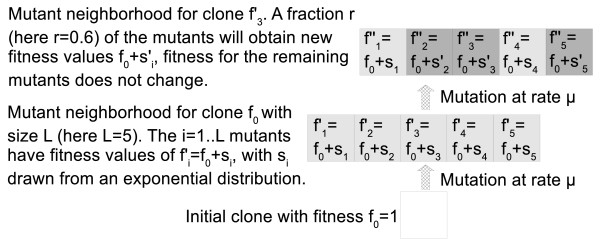
**Schematic of the computational model**. Each clone is characterized by its fitness value, and has a neighborhood of 1-step mutants of size *L*. When a mutation occurs, a random mutant from this neighborhood is selected. For instance in the figure, the initial clone with fitness *f*_0 _gives rise to the mutant . This mutant has access to another set of *L *mutants, etc. The fitness of each mutant is *f*_0 _+ *s*_*i*_, where the *s*_*i *_are drawn from an exponential distribution. This can lead to mutants reaching fitness peaks. If for instance all  have lower fitness than the mutant , this mutant is considered to have reached a local peak. In the figure, three out of five fitness values of the 1-step neighborhood change as the clone mutates from *f*_0 _to  (dark gray blocks). This corresponds to a value of *r *= 0.6 for the parameter which defines the amount of ruggedness of the fitness landscape (see text).

Every clone is assigned a 1-step neighborhood of *L *mutants that can be reached by a single mutation. The ancestral clone is assigned a fitness value of 1 and the fitness values for the *L *mutants are 1 + *s*_*i*_, with values for *s*_*i *_drawn from an exponential distribution *p*(*s*) = *αe*^*-αs *^[19-23]. This mimics a situation where a population finds itself in a new environment to which it is ill adapted and starts an adaptive evolutionary walk towards increased fitness. When a clone divides, one of the *L *1-step mutants is generated with a probability *μ*. Whenever a new mutant is generated, it obtains its own neighborhood of *L *1-step mutants with fitness values of 1 + *s*_*i *_(Figure 2). Note that while all *L *mutants in the 1-step neighborhood of the ancestral clone have higher fitness (1 + *s*_*i *_> 1), the 1-step neighborhood of a newly created clone with fitness 1 + *s*_*i *_can generate mutants that have higher or lower fitness, depending on the randomly chosen values of *s*_*i *_for these mutants. This generates a potentially rugged fitness landscape with multiple peaks. *L *can be interpreted as the mutational target size of selection, or more generally the number of possible beneficial mutations from a given starting genotype.

By adjusting the rules for how the *L*-mutant neighborhood is chosen, we can tune the fitness landscape from one that is completely smooth to one that is completely rugged. For the smooth landscape, each newly created mutant is assigned a mutant neighborhood that is identical to that of the ancestral strain. In other words, the mutation does not alter the fitness effects of any subsequent mutations that might be obtained. Under these conditions there is a single fitness peak. At the other extreme, for a completely rugged landscape, every new mutant is assigned an entirely new L-mutant neighborhood with values for the fitness effect of each new mutation re-sampled from *p*(*s*). This means that a new mutation changes the fitness effects of all other possible mutations. In this scenario there is no correlation between the fitness effects of the *L*-mutants from a parent clone and those available to its mutant offspring. By changing the fraction, *r*, of the *L *sites that are replaced, we can tune the amount of ruggedness of the landscape from smooth (*r *= 0) to completely rugged (*r *= 1). By considering a broad range of values for *r *and *L*, we can explore a range of scenarios in order to identify conditions where changes in parameters lead to qualitative changes in adaptation. Figure 2 schematically shows an example for *r *= 0.6.

While the ability to tune between smooth and rugged fitness landscapes is analogous to that of previously studied NK-models and their variants [24-31] there is a crucial difference between NK-models and our model. In NK-models, there is typically no explicit distinction between the stochastic trajectory of a given population and the fitness landscape upon which adaptation occurs. This means that for every simulation, not only does the adaptive trajectory of the evolving population change, but so does the fitness landscape itself. We sought to disentangle these two sources of variation. Our simulation was designed to ensure that populations of different sizes, for each parameter set, experienced the same fitness landscape. In order to determine the generality of our results across a range of fitness landscapes, we sampled a total of 50 distinct fitness landscapes and then for every fitness landscape, we study 100 evolutionary trajectories for populations of different sizes. On each landscape, this approach was designed to mimic the evolution of bacterial populations in laboratory experiments, where differences in evolutionary trajectories across replicate bacterial lineages are most often due to the stochastic nature of mutations occurring in a single *fixed *fitness landscape, rather than to differences arising from the fact that different lineages experience distinct ecological or genetic conditions [32,33]. The simulations are implemented in Matlab R2007a (The Mathworks), the code is available from the authors. Table 1 summarizes the model parameters and values used throughout the paper.

**Table 1:** Model parameters.

**symbol**	**meaning**	**values**
*N*_0_	initial size of population	10^2^, 10^4^, 10^6^
*L*	size of mutant neighborhood (number of accessible 1-step mutants)	5, 50, 500
*r*	fraction of mutant neighborhood that is changed (ruggedness of fitness landscape)	0.1, 0.5, 1
*μ*	beneficial mutation rate per replication	10^-6^
*D*	number of divisions per growth cycle	10
*α*	distribution of fitness effects	30

Beneficial mutation rate, divisions per growth cycle and distribution of fitness effects were chosen in line with values reported in [16,14] and [20] respectively. The other parameters were chosen to explore the evolution of populations for a wide range of scenarios, as described in detail in the main text.

## Results

### Adaptation on a rugged landscape

On a smooth fitness landscape, all populations will eventually reach the sole fitness peak, with the larger populations doing so more rapidly. However, this can change during adaptation on a rugged landscape, as explained above. Here, large populations are expected to evolve almost deterministically. This allows them to quickly reach the highest *local *fitness peak, where, if asexual, they can become trapped. In contrast, a smaller population size allows for more stochastic trajectories on the fitness landscape, and this can occasionally lead to higher fitness peaks. The transition from more stochastic to more deterministic trajectories occurs as the mutation supply rate, *S*, becomes so large that a population is able to completely sample all possible 1-step beneficial mutations, i.e. if *S ≈ L *[34]. The mutation supply rate is the product of mutation rate and effective population size, *S *= *N*_*e*_*μ*. For the three initial population sizes we consider here, *N*_0 _= 10^2^, 10^4 ^and 10^6^, an effective population size given by *N*_*e *_≈ *DN*_0 _[1], and mutation rate *μ *= 10^-6^, the mutation supply rates are *S*_*s *_= 0.001, *S*_*m *_= 0.1 and *S*_*l *_= 10 for the small, medium and large populations respectively. We initially choose the size of the 1-step neighborhood to be *L *= 50, which means *S*_*l *_≈ *L*, *S*_*m *_<*L *and *S*_*s *_« *L*. Thus we expect the large population to evolve mostly deterministically, while the medium population is expected to evolve somewhat slower, but with the potential of reaching higher fitness peaks. Because the small populations have *S*_*s *_« 1, they are expected to operate in the strong selection weak mutation limit, where evolution will be slow because it is limited by the infrequent creation of beneficial mutations [35,36].

**Figure 3:**
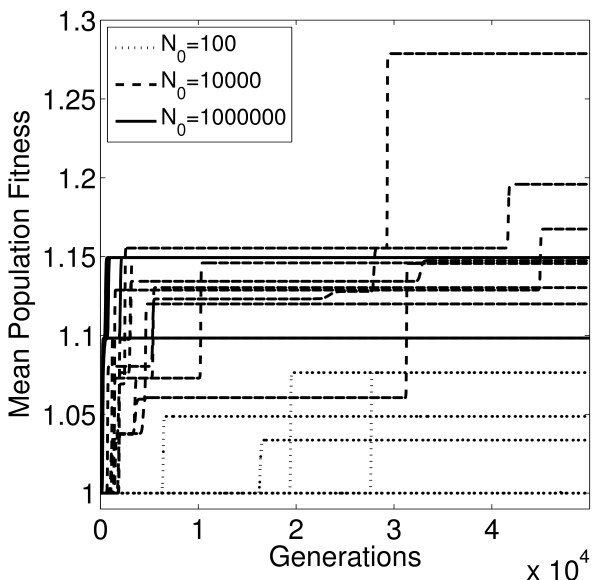
**Evolution on a rugged landscape**. Mean population fitness for the first 10 of the 100 evolutionary trajectories for each population size as a function of the number of generations elapsed. All evolutionary trajectories occur on the same fitness landscape (landscape 25, see Figure 5). Population fitness is defined as the mean of the fitness values of each individual bacterium in the population. Parameter values as given in Table 1, with *r *= 1 and *L *= 50.

**Figure 4:**
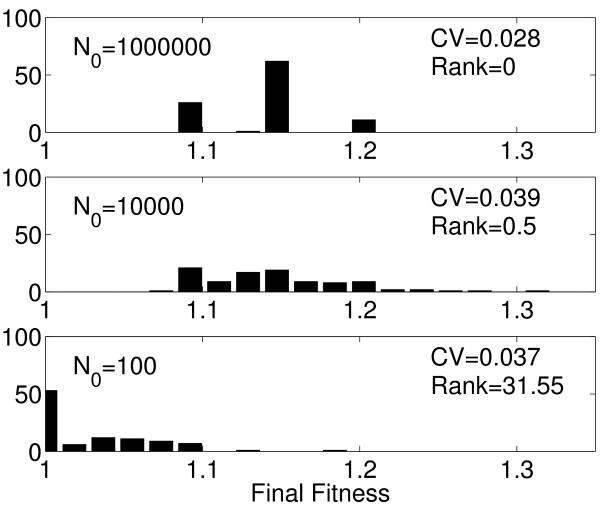
**Evolution on a rugged landscape**. Distribution of final population fitness of the 100 trajectories for the different population sizes. Parameter values as given in Table 1, with *r *= 1 and *L *= 50. CV = coefficient of variation, defined as the standard deviation divided by the mean of the 100 population fitness values at the end of the simulation. For an explanation of rank, see text.

For each of the three population sizes, we simulated 100 evolutionary trajectories for 50 different rugged fitness landscapes (*r *= 1). An example of the results for a single landscape is shown in Figures 3 and 4. Figure 3 shows sample trajectories for the different population sizes. The figure indicates that, as expected, the large populations evolve most rapidly. However, on this fitness landscape, these large populations typically become trapped on a few local fitness peaks which rapidly causes their adaptive ascent to cease. In contrast, the medium sized populations evolve somewhat more slowly but reach a more diverse set of fitness peaks, several of which are higher than the local fitness peaks reached by most of the large populations. While almost all of the large and medium populations have reached fitness peaks, most of the small populations have not. This can be seen by quantifying the rank of the most frequent clone in each population at the termination of the simulation. Rank for a given clone is defined as the number of accessible beneficial mutations in the 1-step neighborhood of this clone [31]. A rank of zero indicates that the mutant has reached a fitness peak and that no 1-step mutations with higher fitness are available. Non-zero values indicate that 1-step beneficial mutations are still available, and consequently that these populations can continue to adapt. The average rank values indicate that the medium and large populations have reached a local peak for nearly all simulations (Figure 4). In contrast, for the small populations the most frequent clone at the end of the simulation is still far from exhausting all available 1-step beneficial mutations, i.e. the small populations are still in the midst of their slow climb towards a fitness peak. This illustrates the trade-off between fast, mostly deterministic adaptation with the potential of becoming stuck on local peaks for large populations, and slower, more stochastic evolutionary trajectories that provide a chance to avoid becoming stuck on local peaks for small populations. An intermediate population size that trades some speed for the ability to reach higher fitness peaks could under such circumstances be optimal. The increased stochasticity in evolutionary trajectories for the small and medium populations is confirmed by the higher coefficient of variation in fitness across replicate populations (Figure 4).

**Figure 5:**
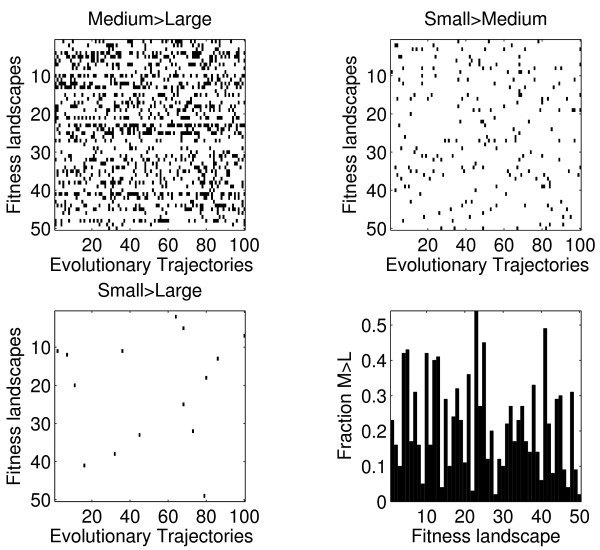
**Evolution on different rugged landscapes (*r *= 1)**. For each of the three population sizes, we simulated 100 different evolutionary walks for each of 50 different fitness landscapes. At the end of every simulation, we recorded the fitness of the most abundant clone. The black squares show those simulations for which the final fitness of this clone for the smaller population exceeds the fitness of this clone for the larger population, as indicated on top of each plot. The bottom right panel shows the fraction of simulations for which the medium populations reach higher fitness than the large populations. The average fraction of M *>*L, S *>*M and S *>*L over all 50 landscapes are 0.218(0.019), 0.038(0.003) and 0.003(0.001), values in parentheses indicate the standard error.

Because these simulations were carried out on 50 independent fitness landscapes we were able to assess the degree to which the result in Figure 4 is general. Figure 5 shows summary results from simulations for all 50 landscapes. For these plots, we record the fitness of the most abundant clone at the end of each evolutionary trajectory. We then compare this fitness value between the different population sizes. We indicate with black those simulations for which the condition indicated on top of each plot is fulfilled. As can be seen, the fraction of small or medium populations that achieve higher fitness than the larger populations depends strongly on the shape of the underlying fitness landscape. For instance, the bottom right panel shows that the fraction of simulations where the fitness of medium sized populations exceeds the fitness of large populations ranges from 0.02 (landscape 28 and 50) to 0.54 (landscape 23). From these results we draw two conclusions. First, we find that populations of smaller size regularly (though not in the majority of cases) attain higher fitness than larger populations. Second, these data indicate that this outcome relies strongly on the detailed shape of the fitness landscape. We now explore in more detail how differing values of the model parameters impact these conclusions.

### The impact of landscape ruggedness

**Figure 6:**
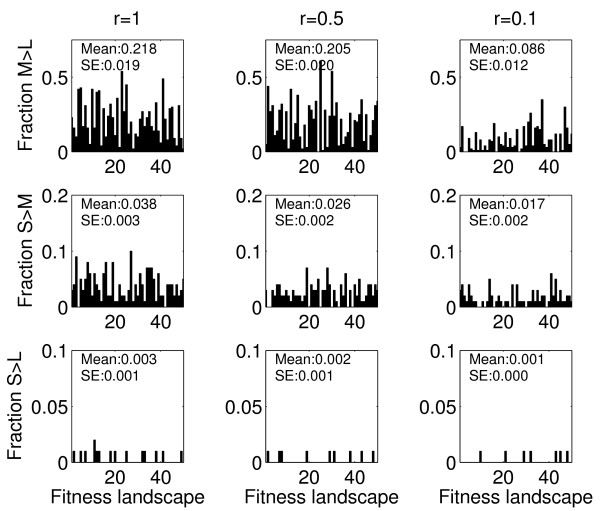
**Fraction of simulations for which small or medium size populations achieve higher fitness than their larger counterparts, for landscapes of different ruggedness (from left to right: r = 1, r = 0.5, r = 0.1)**. The mean and standard error over all fitness landscapes is also shown. Other values as given in Table 1, with *L *= 50.

The previous section showed that on rugged landscapes, population size and fitness landscape architecture strongly interact to influence the dynamics of adaptive trajectories. While empirically characterized fitness landscapes can indeed have multiple peaks, the amount of ruggedness is largely unknown [37-39]. In all likelihood, some landscapes will be simple ones characterized by few peaks, while others will have multiple local fitness peaks. To address the impact of landscape ruggedness, we now change the parameter *r *to tune the ruggedness of the fitness landscape, and examine how it affects the interaction between population size and adaptive processes. As Figure 6 shows, for a less rugged landscape (*r *= 0.5), populations of intermediate size retain their ability to sample the fitness landscape more broadly and to reach higher fitness peaks than large populations. However, as the landscape becomes smoother (*r *= 0.1), this advantage disappears. This is expected, since for a completely smooth landscape (*r *= 0), there is only a single globally optimum peak which would be reached by all populations eventually, simply more rapidly by the larger ones. The small populations are less affected by the change in ruggedness because they are still far away from any peak on which they could become stuck. This is confirmed by the mean final ranks (averaged over all landscapes and trajectories, see Table 2) which again indicate that most of the medium and large populations have reached peaks, while the small ones have not. Coefficients of variation in final fitness for different trajectories (also averaged over all landscapes) are consistently higher for the smaller populations, due to the more stochastic evolutionary trajectories taken by those populations (Table 2). As expected, there is an overall trend for the coefficient of variation to decrease as the landscape becomes less rugged.

**Table 2:** Rank/CV for different population sizes.

**Scenario**	**Population Size**		
Changing ruggedness	Small	medium	Large
*L *= 50, *r *= 1	29(0.42)/0.047(0.001)	0.42(0.02)/0.039(0.001)	0.01(0.01)/0.013(0.002)
*L *= 50, *r *= 0.5	30(0.35)/0.045(0.002)	0.37(0.02)/0.034(0.001)	0.002(0.001)/0.009(0.002)
*L *= 50, *r *= 0.1	30(0.36)/0.045(0.002)	0.36(0.03)/0.02(0.001)	0(0)/0.005(0.001)

Changing mutant neighborhood			
*L *= 5, *r *= 1	3(0.1)/0.034(0.002)	0.04(0.01)/0.026(0.002)	0(0)/0.012(0.002)
*L *= 50, *r *= 1	29(0.4)/0.047(0.001)	0.42(0.02)/0.039(0.001)	0.01(0.01)/0.013(0.002)
*L *= 500, *r *= 1	294(2.8)/0.047(0.001)	3.8(0.09)/0.044(0.001)	0.03(0.01)/0.031(0.001)

Changing mutation rate			
*μ *= 0.5/*N*_*e*_	0.074(0.007)/0.037(0.002)	0.083(0.01)/0.035(0.002)	0.094(0.012)/0.034(0.002)

Rank is the mean for all landscapes and trajectories, CV is the mean CV over all different landscapes. Values in parentheses are standard errors for the sample of 50 landscapes.

### The impact of the size of the mutant neighborhood

**Figure 7:**
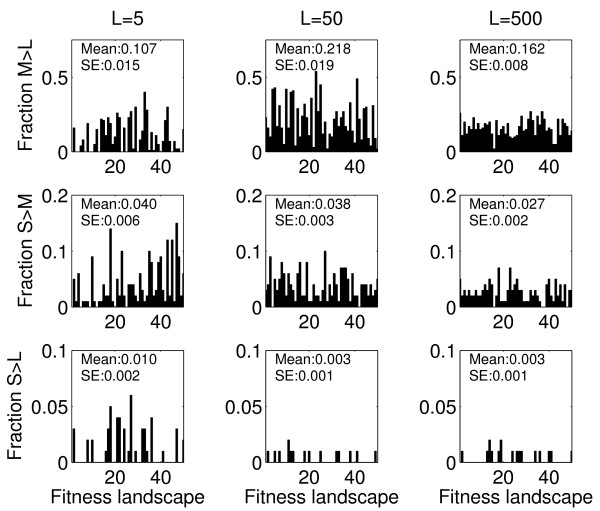
**Fraction of simulations for which small or medium size populations achieve higher fitness than their larger counterparts, for different sizes of the mutant neighborhood (from left to right: L = 5, L = 50, L = 500)**. Parameter values as given in Table 1, with *r *= 1.

In our simulation, *L *represents the size of the one-step mutant neighborhood, i.e. the number of mutants that a clone can reach. As explained above, the relation between *L *and the mutation supply rate, *S*, will determine if evolution occurs in a more deterministic or more stochastic manner. As *L *decreases, a population with a given mutation supply rate is more likely to follow a more deterministic trajectory, while increased *L *leads to more stochastic trajectories. Figure 7 and Table 2 show this to be the case.

For instance for *L *= 5, the medium populations have a reduced amount of stochasticity and are more likely to have reached a (local) peak, compared with the *L *= 50 situation (see Rank and CV in Table 2). This results in a lower fraction of populations that reach fitness higher than that of the large population (compare Figure 7 top row *L *= 5 with *L *= 50). For small *L*, the small populations are less disadvantaged in terms of adaptive "speed" and are able to more frequently, although still quite rarely overall, reach higher fitness peaks than larger populations (compare Figure 7 bottom row *L *= 5 with *L *= 50).

For the large (*L *= 500) scenario, evolution for the large population becomes markedly more stochastic (see CV in Table 2), leading to a broader exploration of the fitness landscape. This results in less frequent instances where the medium populations reach higher fitness than the large populations (compare Figure 7 top row *L *= 50 with *L *= 500). This supports the intuitive understanding that if more beneficial mutations are accessible, the population size that optimizes the trade-off between the speed of adaptation and the magnitude of the adaptive response shifts towards larger populations. Indeed, in the limit of *L *→ ∞, every clone has access to all possible other mutants, in essence reducing the system to a smooth landscape on which the large populations are always favored [30,34].

### Mutation rates versus population sizes

Above, we explained how the relation of the mutation supply rate, *S*, and the mutant neighborhood, *L*, are relevant for determining whether adaptation tends to be dominated by stochastic or deterministic change. The mutation supply rate is the product of population size and mutation rate. It is known that population size and mutation rate can have differential effects on the evolutionary dynamics [13,40,41]. For example, fixation times are faster in smaller populations, even though mutations arise less often.

**Figure 8:**
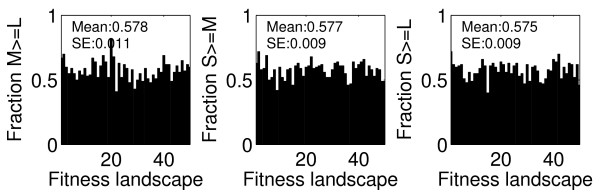
**Fraction of simulations for which small or medium size populations achieve higher fitness than their larger counterparts**. The mutation rate is adjusted such that all populations have a fixed mutation supply rate *S *= *μN*_*e *_= 0.5 (i.e.*μ *= 5 × 10^-4^, 5 × 10^-6 ^and 5 × 10^-8 ^for the small, medium and large populations respectively). Parameter values as given in Table 1, with *r *= 1, *L *= 50.

We therefore examined how changing mutation rate and population size, while keeping mutation supply rate fixed, influenced the results of our simulations. As Figure 8 shows, for a fixed mutation supply rate, smaller populations with higher mutation rates seem to have a slight advantage. The final average rank values and CV for the different populations are rather similar, with a slight trend towards increasing rank and decreasing CV as population size increases (Table 2). This suggests that for a fixed mutation supply rate, smaller populations evolve both somewhat faster and somewhat more stochastically. The reason for these results is likely a consequence of clonal interference [11,12,16,42,43]. If populations are large, several beneficial clones compete against each other, with the largest one likely winning and becoming fixed. This would lead to more deterministic adaptive trajectories compared to smaller populations [41]. Therefore, at the same mutation supply rate, smaller populations with higher mutation rates could be favored over larger populations with lower mutation rates.

## Discussion and Conclusion

It is generally accepted that large populations will tend to evolve more rapidly than smaller ones. This is caused by two related factors. First large populations have an increased supply of beneficial mutations each generation, which decreases the waiting time for new advantageous mutations. Second, large populations have increased access to mutations that confer large benefits. These factors imply that larger populations gain an advantage by taking larger adaptive steps during population evolution. However, as we have shown in a previous study [14], sometimes smaller populations can reach higher levels of fitness. Here, we have explored this phenomenon in more detail. We found that while large populations evolve faster on both smooth and rugged landscapes, on the latter there can be a trade-off between speed and the potential to reach high fitness peaks. Because large populations tend to fix the most advantageous mutations first and thereby follow a very limited set of adaptive trajectories, they have a tendency to become trapped on local fitness peaks. In contrast, smaller populations become fixed for a wider range of possible beneficial mutations which leads to increased variation in adaptive trajectories across populations and allows some populations to avoid becoming trapped on local peaks. However, the potential to reach higher fitness peaks can come at the cost of a slower speed in adaptation. The optimal population size therefore likely depends on the relative importance of speed versus final fitness.

We further showed that for a rather smooth fitness landscape, there is no advantage in following more stochastic adaptive trajectories; however, even an intermediate amount of ruggedness can be sufficient to occasionally favor more stochastically evolving populations of smaller size. Experimental studies suggest that at least some amount of ruggedness is present in natural situations [38,44-47]. We also showed that when the size of the mutational target under selection is very large or very small, the system converges to an effectively smooth landscape where large populations are favored.

Lastly, we found some evidence that for a fixed mutation supply rate, small populations evolved more rapidly and more stochastically, which allowed them to reach higher fitness compared to larger populations in a majority of simulations. We suggest that this can be attributed to clonal interference acting in larger populations, which limits the amount of within population variation and can retard the rate of adaptation [16]. To keep the mutation supply rate constant, it was necessary to increase the mutation rate for the small populations. That this tended to confer an advantage may imply that small populations, such as bacterial pathogens at or following the bottleneck during transmission, may benefit by adopting a transient mutator phenotype in order to successfully colonize new hosts. An important caveat to this is that if the mutation load increases with mutation rate, with an associated increase in genetic drift during bottleneck transmission, a mutator strategy would carry a profound cost, both for individual populations and descendant lineages in separate hosts [48].

Although medium and small populations can exceed the fitness of larger populations, we note that this outcome does not occur in all, or even most, simulations. More important, the degree to which this result is realized is highly dependent upon underlying landscape architecture. For example, as is most clearly evident in Figure 5, there is considerable variation in the fraction of cases where populations of medium size exceed the fitness of large populations, with a broad range from 0.02 to 0.54. Several features of the fitness landscape influence the potential outcome of the adaptive walks. First, if the closest fitness peak is a global peak, medium and small populations would fail to capitalize on their greater searching ability. This would also apply if the local peak is the highest peak within a certain "radius" of the starting location in the fitness landscape, since a far away peak might never be reached by any of the populations. Second, the difference between the global peak and accessible local peaks may be negligible, in which case differences in adaptive magnitude across populations of different sizes will be similarly small. Finally, the global peak may not be accessible at all, in which case the smaller populations will again fail to capitalize upon their potential search advantages.

As with any model, we have made several simplifications. For instance we excluded death of bacteria and only allowed the loss of novel mutants to occur through stochastic loss during sampling via serial dilution. The inclusion of stochastic drift [49] would likely not change the bulk of our results, but it might impact some of the details, especially for our small population size with *N*_0 _= 100.

A second simplification is our exclusive focus on asexual populations. A number of studies have shown that the incorporation of recombination can help to overcome clonal interference or can help populations to more easily escape from local fitness peaks [50-53], though recombination might not be always beneficial [54]. Extending our model to allow for recombination is a focus of future studies and will allow us to understand how recombination may help large populations to avoid becoming trapped upon local fitness peaks.

We used our simulation to study populations that ranged in size over 4 orders of magnitude. In this range, we found that our large populations exhibited clonal interference and very rarely escaped from local fitness peaks. However, a number of recent studies suggest that if the population size is large enough, the impact of clonal interference might be reduced [43,55-57]. Additionally, very large populations are expected to more easily escape from local fitness peaks [58-62]. For the combination of population size and severity of bottleneck we used in our simulations, we found that deleterious mutants were removed from the population most of the time before they could reach appreciable frequencies and lead to compensatory mutations. This may suggest that for evolution through growth-bottleneck cycles (which applies not only to laboratory situations, but is likely also applicable to many pathogens), the bottleneck size interacts strongly with the population size and other parameters to determine the dynamics of the evolutionary process [17,18,63]. Further investigation of the interactions of population size, landscape ruggedness and mutation rate with bottleneck size, and the importance of different types of mutations during growth-bottleneck cycles [64] deserves further study.

In summary, we have shown that for asexual populations evolving on rugged fitness landscapes, there can be a trade-off between speed of adaptation and the attainable fitness, which strongly depends on the underlying fitness landscape. This suggests that the optimal population size likely depends on both the details of the fitness landscape and the relative importance of speed versus final fitness.

## Authors' contributions

AH and DR conceived of the study. AH wrote and analyzed the simulations. AH and DR wrote the manuscript. Both authors read and approved the final manuscript.
